# Advances in Implant Surface Technology: A Biological Perspective

**DOI:** 10.7759/cureus.78264

**Published:** 2025-01-30

**Authors:** Shristi Yadav, Anjali Nath, Reshma Suresh, Arun Kurumathur Vasudevan, Biju Balakrishnan, Maya Rajan Peter

**Affiliations:** 1 Periodontics, Amrita School of Dentistry, Amrita Vishwa Vidyapeetham, Kochi, IND

**Keywords:** bioactive coatings, dental implants, osseointegration, stability, surface engineering

## Abstract

The present review provides comprehensive information about recent developments in surface modification techniques directed to enhance osseointegration. Through an in-depth analysis of key molecular pathways and regulatory factors involved in bone biology, including sclerostin, Wnt signaling, bone morphogenetic protein-2 (BMP-2), and fibroblast growth factor-2 (FGF-2), the review explores how these advancements contribute to promoting osteogenesis, bone healing, and implant stability. By synthesizing findings from preclinical and clinical studies, the manuscript elucidates the potential of surface modification technologies to address challenges in implant dentistry, offering valuable insights for researchers, clinicians, and industry professionals striving to optimize implant success rates and patient outcomes.

## Introduction and background

The loss of a tooth due to injury or disease can result in complications such as accelerated bone resorption, difficulties in speech, and discomfort during mastication. Implant dentistry represents a significant advancement in dental care, fundamentally altering the methodology used to replace missing teeth [1,2]. Dental implants have garnered widespread acceptance and success in this context, improving oral health and overall quality of life. The extensive history of dental implants indicates their sustained success and reliability [3].

Osseointegration plays a pivotal role in creating a stable foundation for dental implants, enabling them to withstand functional forces like chewing and speaking, especially in dental and orthopedic applications. Without this robust connection, implants risk failure under normal physiological loads [4].

The concept of primary stability is integral to successful osseointegration. Achieved at the time of implant placement, primary stability refers to the initial stability derived from the precise positioning and optimal contact of the implant within the bone. This immediate fit is essential for the implant’s initial anchorage and is a precursor to subsequent biological integration. Techniques such as insertion torque measurement and resonance frequency analysis are commonly employed to evaluate primary stability at the time of placement [5].

As osseointegration progresses, the implant achieves secondary stability, marked by enhanced mechanical and biological integration with the surrounding bone. This phase occurs during the healing period and reflects the dynamic process of bone remodeling and adaptation around the implant. Secondary stability is typically assessed postoperatively, following the osseointegration period, using diagnostic methods such as periotest analysis, resonance frequency analysis, or radiographic evaluation. Together, primary and secondary stability ensure the long-term success and functionality of dental implants, underscoring the importance of careful surgical planning and postoperative monitoring [5].

Osseointegration is a pivotal process in dental implantology, ensuring implants securely integrate into the patient's bone to endure functional loads. Surface engineering emerges as a crucial strategy to enhance the success and durability of osseointegrated implants [6]. While titanium and its alloys are widely utilized for their exceptional properties, their bioinert nature presents challenges in directly binding to bone tissue post-implantation [7]. Therefore, the surface engineering of osseointegrated implants is crucial to their success and durability. This field of surface modification technology has evolved into a highly interdisciplinary domain, primarily focused on improving the osseointegration of titanium implants [8]. These modifications target various aspects of implant performance, resulting in a spectrum of favorable outcomes. As a result of bioactive surface modifications on implant surfaces, significant improvements in peri-implant bone formation and osseointegration have been demonstrated, particularly when applied during the initial phases of healing [9].

Surface properties play a crucial role in determining the effectiveness of implants in integrating with surrounding bone tissue. Titanium has traditionally been a preferred material for medical implants; however, the prolonged recovery period following dental implantation presents a significant challenge, heightening the risk of early implant failure due to inadequate osseointegration. Early implant failure is primarily caused by inadequate osseointegration. Factors contributing to this include poor bone quality or quantity, improper surgical technique, contamination or infection at the implant site, and excessive micromovements during the healing phase.

In response to this challenge, researchers have focused on enhancing the biological activity of implant materials to accelerate osseointegration within a reduced timeframe. The interface between the implant and tissue represents a dynamic and complex environment influenced by various factors, including the properties of the biomaterial, biocompatibility, mechanical considerations, and biological processes [10]. Consequently, investigators have developed and examined an array of materials, coatings, and surface treatments to optimize the biomechanical properties of the implant-bone interface.

## Review

Need for bioactive surface coating

Titanium and its alloys are among the most popular implant materials due to their excellent ability to integrate with bone without forming a fibrous interface layer. However, they have certain drawbacks. For instance, metal ions released from titanium implants have been detected in adjacent tissues, potentially causing local irritation [11]. Consequently, the chemical composition, mechanical properties, and surface topography of the implant surface significantly influence cellular and tissue reactions. Recent research by Albrektsson et al. suggests that commercially pure titanium may trigger an immune-modulated inflammatory response when placed in living tissues, raising concerns about its potential impact on hard tissue integration [12].

The original Branemark implant, a machined screw with low surface roughness, was long considered the benchmark [13]. However, experimental studies have shown that implants with titanium plasma-sprayed surfaces achieve greater bone-to-implant contact than smooth-surfaced titanium implants [14]. Another surface modification technique, anodic oxidation, promotes the formation of a porous titanium oxide layer. Histological analyses from animal studies and human implant retrievals have demonstrated that this modification results in strong bone-to-implant interlocking [15].

Surface roughening techniques like grit blasting, acid etching, and sandblasting with large grit, followed by acid etching, along with coating methods such as calcium phosphate and hydroxyapatite, are commonly employed to enhance the bioactivity of implant surfaces. However, these methods may not always ensure optimal biocompatibility, as the high temperatures involved can interfere with the integration of biological agents. Tailoring surface coatings to meet individual patient needs can effectively improve implant bioactivity. Furthermore, optimizing implant surface properties through topographical and chemical modifications has garnered significant interest, particularly with biologically active agents [16].

Conventional implant materials, including titanium and titanium alloys (e.g., Ti-6Al-4V) and zirconia, benefit from such surface modifications. Among the various techniques, coatings with bioactive materials such as hydroxyapatite, arginyl-glycyl-aspartic acid (RGD) peptides, or bone morphogenetic protein-2 (BMP-2) are regarded as superior. These bioactive coatings foster direct chemical bonding with bone tissue, enhancing osseointegration, improving implant stability, and reducing healing time. Additionally, they contribute to better mechanical integration, minimize the risk of implant rejection, and ensure improved clinical outcomes, positioning them as the preferred choice over conventional methods [17].

The use of bioactive coatings on dental implants offers substantial advantages in achieving superior osseointegration. These coatings establish a chemical bond with surrounding bone tissue, facilitating integration. Moreover, bioactive coatings are specifically designed to be highly biocompatible, ensuring tolerance by the body without triggering toxic or inflammatory responses. This characteristic minimizes the likelihood of rejection or adverse reactions, creating an optimal environment for tissue healing and integration [18].

Conventional implant materials, including titanium, titanium alloys (such as Ti-6Al-4V), and zirconia, are widely used in dental and orthopedic implants due to their biocompatibility, mechanical strength, and corrosion resistance. However, unlike these conventional materials, bioactive coatings are less likely to elicit a foreign body response. Their composition closely resembles natural bone minerals, making them more compatible with the body’s biological processes. Bioactive coatings also enable direct bonding with living bone tissue, known as bioactive bonding or bioactive fixation. This enhances implant stability, promotes longevity, aids in load transfer, and ensures even stress distribution, significantly reducing the risk of implant failure [18].

**Figure 1:**
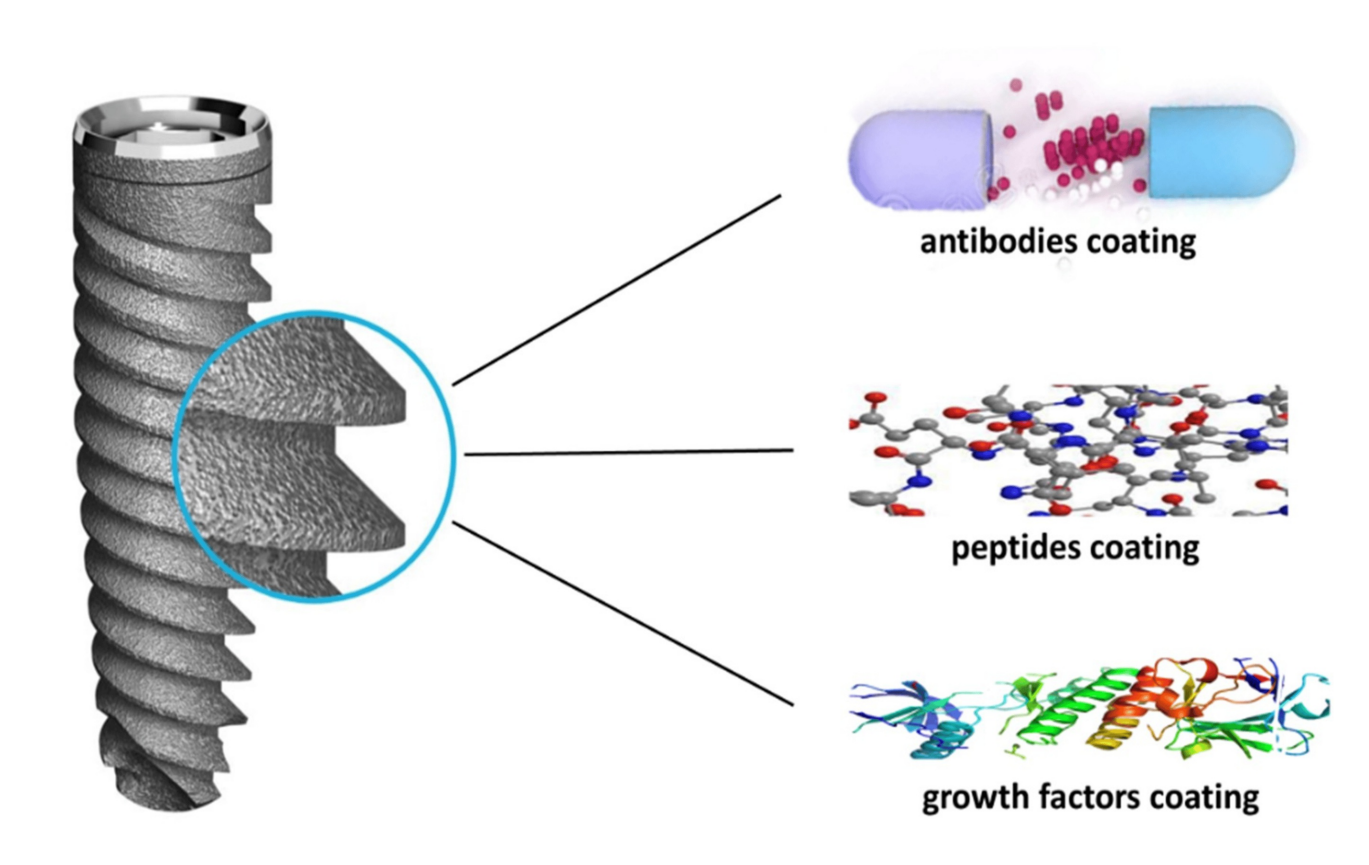
Various materials used for bioactive coating of dental implants to enhance osseointegration Image Credit: Dr. Anjali Nath

The superior osseointegration achieved through bioactive coatings significantly improves dental implants' long-term stability and success. Patients benefit from improved implant retention, functionality, and aesthetics, resulting in greater dental restoration satisfaction. Numerous in vitro studies have demonstrated that peptide-modified surfaces significantly enhance cell attachment, resulting in stronger adhesion than unmodified surfaces [19]. This enhancement is primarily attributed to specific binding interactions between peptides and cell surface receptors. Various materials used for the bioactive coating of dental implants to enhance osseointegration are illustrated in Figure 1.

RGD-coated implants

Among peptide motifs, RGD is the most widely recognized motif for mediating cell adhesion to the extracellular matrix. It is conserved across various species, ranging from *Drosophila* to humans. The RGD sequence serves as a key adhesive motif in numerous proteins, including fibronectin, thrombospondin, and osteopontin, which are components of the bone extracellular matrix. This motif regulates various cellular responses, from short-term functions such as adhesion to long-term events like protein secretion. When coupled with integrins-receptors that recognize cell adhesion molecules-RGD-containing proteins facilitate essential cellular interactions [20].

Several RGD peptides have demonstrated the capacity to enhance cellular responses to materials with limited bioactivity, particularly in tissues undergoing repair. By manipulating the density, patterning, structure, and orientation of synthetic RGD peptides, it is possible to elicit cell responses beyond those triggered by native matrix molecules. This level of control presents significant potential for advancing tissue-engineered constructs. Functionalizing implant surfaces with immobilized RGD peptides has been shown to improve bioactivity and promote osteogenic differentiation of osteoblasts, thereby enhancing their adhesion to titanium surfaces [21].

Coating dental implant surfaces with RGD peptides significantly boosts osteoblast adhesion and accelerates osseointegration. Bone-active coatings can also be developed by directly incorporating bone extracellular matrix proteins onto the implant surface. These proteins interact with integrins, activating multiple intracellular signaling pathways critical for cellular adhesion and integration.

**Figure 2:**
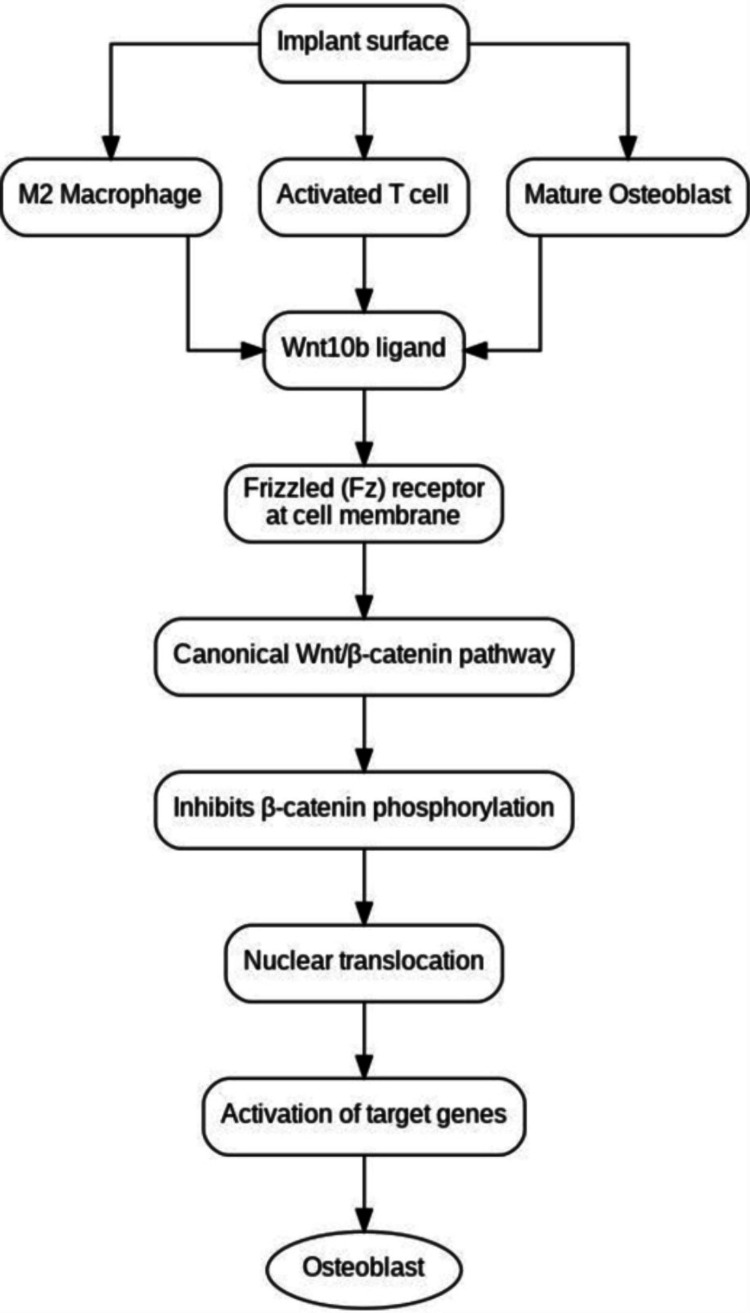
Canonical Wnt pathway and osteoblast differentiation around dental implants Image Credit: Dr. Anjali Nath

Various studies have substantiated the critical role of the RGD motif during the early stages of cell-biomaterial interaction at the implant interface [22]. Soluble RGD peptides are known to impair cell attachment, with the extent of this effect varying depending on the surface composition of the implant. Empirical evidence indicates that RGD peptides expedite tissue binding to implant surfaces, as demonstrated by their ability to enhance cellular adhesion strength during the crucial postoperative period significantly. This accelerated healing process and improved initial osseointegration are pivotal for the long-term success of implant integration (Figure 2). The studies investigating the effects of RGD on osseointegration are summarized in Table 1.

**Table 1:** Studies providing evidence for surface modifications RGD: arginyl-glycyl-aspartic acid, Ti-6Al-4V: titanium alloy composed of 90% titanium, 6% aluminum, and 4% vanadium, Scl-Ab: sclerostin antibody, ZOL: zoledronic acid, BMP-2: bone morphogenetic protein-2, rhBMP-2: recombinant human bone morphogenetic protein-2, FGF: fibroblast growth factor, FGF-2: fibroblast growth factor-2, TiO₂: titanium dioxide, 3D: three dimensional

Author	Conclusion
Yang et al., 2009 [23]	RGD-coated porous implants showed increased peri-implant bone formation, higher removal torque values, and improved bone-bonding ability.
Alkhodary, 2014 [24]	Tri-peptide RGD coating on custom-made titanium (Ti-6Al-4V) implants under mandibular overdentures demonstrated enhanced bone density and reduced vertical bone loss after five years.
Yu et al., 2018 [25]	Systemic administration of Scl-Ab enhanced maxillary bone osseointegration and regeneration around implants.
Korn et al., 2019 [26]	Combined ZOL and sclerostin antibody treatment improved bone-implant contact, cancellous bone mineral density, and bone volume/tissue volume.
Couto et al., 2023 [27]	Reviewed evidence showing that Scl-Ab enhanced peri-implant bone formation and osseointegration.
Teng et al., 2016 [28]	Implants coated with BMP-2 exhibited superior bone quality due to increased mineralization and osseointegration, facilitated by greater contact areas between the implant and adjacent bones.
Lyu and Lee, 2020 [29]	Hydrogel composite loaded with rhBMP-2 promoted osteogenesis around dental implants within bone defects, enhancing osseointegration.
Eawsakul et al., 2021 [30]	BMP-2 coating on titanium demonstrated uniformity, biocompatibility, and robust stability, fostering osteoblast proliferation.
Nagayasu-Tanaka et al., 2017 [31]	Formation of new bone around dental implants is facilitated by FGF, enhancing implant stability through improved osseointegration.
Aragoneses et al., 2021 [32]	FGF-2 accelerates bone mineralization at the implant interface, enhancing biofunctionalization and osseointegration. Carboxyethylphosphonic acid coating facilitates FGF-2 immobilization as a biomimetic method.
Sidhu et al., 2023 [33]	Discusses advancements in bio-implants, including resorbable bone wax, anti-cancer TiO₂ coatings, nanostructured alloys, and 3D-printed patient-specific implants. Highlights commercialization challenges and the need for interdisciplinary research to enhance healthcare.
Dua et al., 2024 [34]	Surface treatment advancements significantly improved osseointegration and clinical outcomes, including traditional methods (acid etching, sandblasting, plasma spraying) and advanced techniques (anodization, hydroxyapatite coatings, laser surface modification, nanotopography).

Sclerostin-antibody coatings

Sclerostin, a glycoprotein consisting of 213 amino acids, is primarily expressed by osteocytes and acts as a mediator in the communication between osteocytes and osteoblasts [35]. Its main function is to negatively regulate bone formation by inhibiting osteoblast differentiation. Genetic studies have shown that individuals with mutations in the SOST gene, which encodes sclerostin, display increased bone density and mass throughout the skeletal system. Conversely, animal studies have demonstrated enhanced bone formation, mass, and strength in SOST-null mice, while overexpression of the SOST gene results in osteopenia. Based on these findings, antibodies targeting sclerostin have been developed to treat bone disorders.

The inhibitory effect of sclerostin on osteoblasts is mediated through its disruption of the Wnt signaling pathway. Sclerostin binds to Wnt co-receptors, LRP5 and LRP6, thereby blocking the pathway [36]. The Wnt signaling pathway is essential for promoting pre-osteoblast proliferation and osteoinduction, which are critical for bone healing. It increases the population of osteoblastic lineage cells by steering mesenchymal stem cells (MSCs) away from differentiation into chondrocytes and adipocytes. Additionally, sclerostin influences the maturation of osteoclasts by modulating RANKL levels in osteoblast receptors (Figure 2). This protein plays a crucial role in skeletal development and bone homeostasis, with abnormalities in Wnt pathway components leading to skeletal anomalies.

In dental implants, the Wnt/β-catenin pathway's role in promoting osteoblastic differentiation is particularly significant for osseointegration. At the implant-bone interface, various cells, including macrophages, T cells, and mature osteoblasts, release the Wnt10b ligand, which directs MSCs toward an osteogenic lineage. Activation of the Wnt/β-catenin pathway within these cells drives osteoblast differentiation and maturation. Enhancing Wnt signaling around implants has been proposed as a potential strategy for reducing implant failure.

Systemic administration of monoclonal sclerostin antibodies has enhanced Wnt signaling, increasing bone formation and strength [37]. This results in improved bone-implant contact, increased bone mass, and enhanced bone performance, even with aging. Although specific clinical studies are still lacking, ongoing research is investigating the therapeutic potential of sclerostin inhibition to improve the performance of titanium implants.

Bone morphogenetic proteins

**Figure 3:**
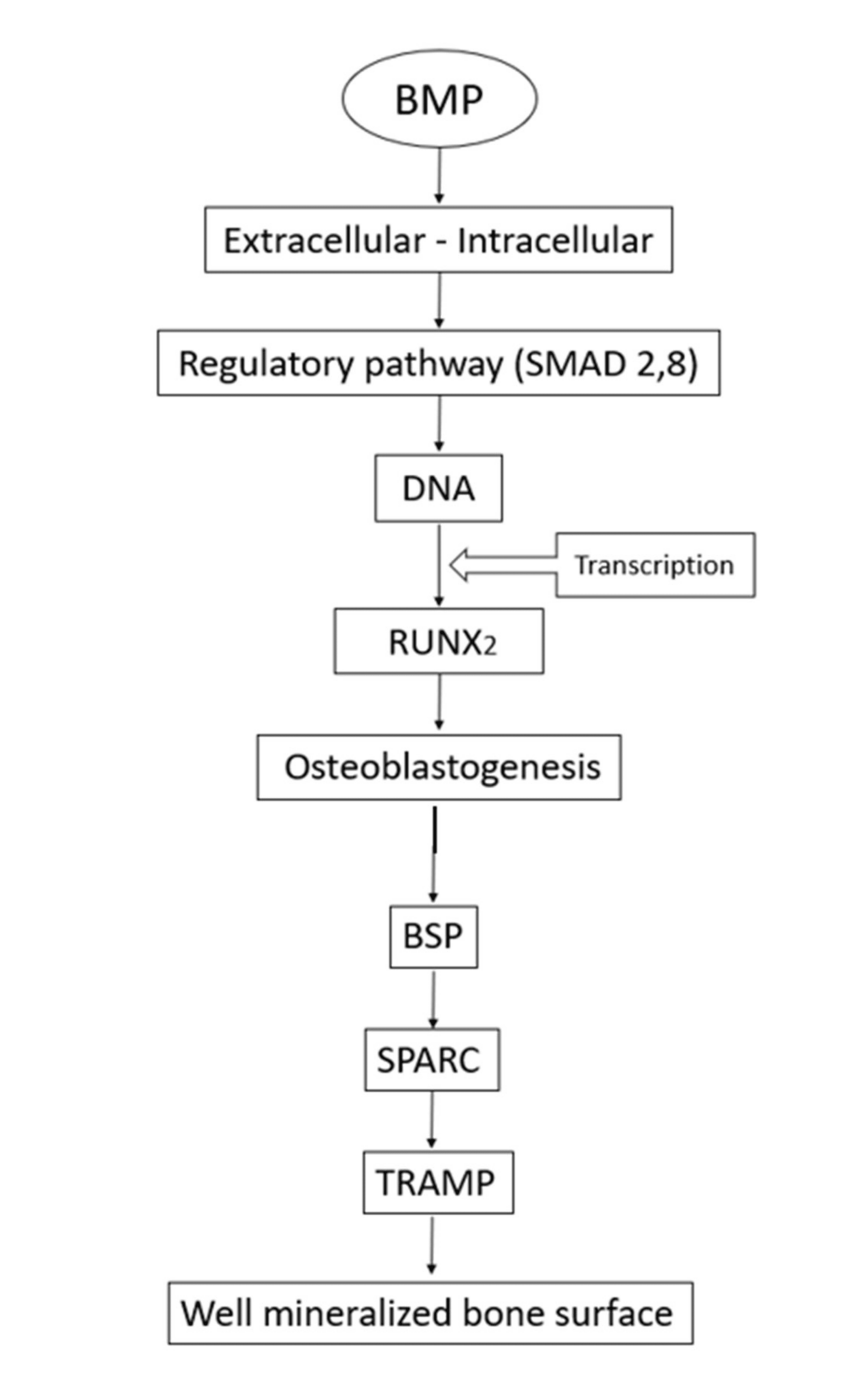
Pathway by which BMP coatings improve bone mineralization around implants BMP: bone morphogenetic protein, SMAD: suppressor of mothers against decapentaplegic, RUNX2: runt-related transcription factor-2, BSP: bone sialoprotein, SPARC: secreted protein acidic and rich in cysteine, TRAMP: tyrosine-rich acidic matrix protein Image Credit: Dr. Anjali Nath

BMPs, members of the TGF-β superfamily, play a crucial role in osteogenesis by promoting the differentiation of osteogenic cells into osteoblasts. These biological factors significantly influence bone formation and mineralization, particularly in the context of implant integration. BMP coatings on implants have been demonstrated to enhance bone mineralization by activating intracellular signaling pathways, most notably the SMAD pathway (Figure 3).

This signaling cascade leads to the activation of osteogenic genes such as RUNX2, promoting osteoblastogenesis and the formation of osteoblasts. Osteoblasts, in turn, synthesize key proteins like BSP, SPARC, and TRAMP, which regulate mineralization and modulate cytokine activity, ultimately resulting in improved mineralized bone surface around dental implants.

Osteoblasts, derived from MSCs, commit to the osteoblast lineage after exposure to BMP-2, emphasizing the pivotal role of BMP-2 in bone formation. BMP-2-containing titanium implants have demonstrated increased density of surrounding bone and better bone-to-implant contact compared to other surface treatments. BMP-2, secreted by osteoblasts, osteocytes, and endothelial cells, binds to BMP receptors on MSCs, initiating osteoblast differentiation and subsequent secretion of the organic matrix of bone. In vivo investigations have shown increased adhesion and proliferation of osteoblastic cells with BMP-2-based coatings, highlighting the potency of BMP-2 as a regulator in osteogenic pathways [38].

Studies comparing different modes of BMP-2 delivery within defined osteoinductive spaces have demonstrated sustained osteogenic activity, particularly with coating-incorporated depots of BMP-2. Additionally, BMP-2-coated surfaces on titanium implants have been found to enhance cell viability and accelerate bone regeneration. The potential benefits of applying BMPs to titanium surfaces include improved patient outcomes and increased implant success by shortening the time needed for implant integration and increasing implant osseointegration.

Fibroblast growth factor-coated implants

Fibroblast growth factor (FGF) is a prominent growth factor renowned for its potential in tissue repair and regeneration. This therapy can rejuvenate various tissues, including skin, blood vessels, muscles, adipose tissue, tendon bones, and cartilage. In addition to proliferating, sustaining, metabolically regulating, morphogenetic, differentiating, and regenerating tissues, FGFs also play a role in the regeneration and repair of damaged tissues. Upon binding with its receptor, FGF initiates a series of reactions culminating in the activation of genes in the nucleus, thereby eliciting cell responses such as division or proliferation. Basic FGF, a member of the FGF family, exhibits particular potency, exerting a proliferative effect on osteoblasts, thereby contributing to increased bone collagen. FGFs are acknowledged as mitogenic factors for cells originating from mesenchymal and neuroectodermal lineages, playing a significant role in angiogenesis during the healing process [39].

Earlier research has demonstrated that FGF-2 facilitates osteogenesis around dental implants and subsequent osseointegration, enhancing implant stability [40]. Thus, using FGF-2 could improve success rates when implant failure is high. Therefore, the topical application of FGF-2 alongside various osteoconductive materials presents a promising avenue for achieving osseointegration with an increased likelihood of success.

Limitations

A proficient coating technique can significantly influence the mechanical properties of dental implants and yield beneficial effects in dental implant applications. However, these techniques have various limitations, such as inadequate adhesion to the substrate, nonuniform layer thickness, and inconsistency in coating composition and crystallinity.

Achieving an optimal balance of wear resistance, corrosion resistance, biocompatibility or bioactivity, and antibacterial activity solely through surface modification technology remains challenging in biomedical implants. Ensuring control over unforeseen side effects is imperative before clinical implementation. Challenges persist in addressing conjugation issues between biofunctional molecules and implant surfaces, particularly during implant installation in clinical settings.

From a commercialization standpoint, several critical questions and challenges about manufacturing, coating approaches, sterilization, packaging, and shelf life of dental implant coatings need resolution. Overcoming these hurdles is vital for the successful market introduction of dental implant coatings. Collaboration among researchers, engineers, manufacturers, regulatory agencies, and clinicians is essential to surmount these obstacles and ensure that coated implants meet stringent quality, safety, and performance standards.

Future advancements

The future of implant research should prioritize the development of surface enhancements that optimize clinical outcomes across various parameters, such as promoting osteogenesis and cellular growth, facilitating soft-tissue attachment, and inhibiting microbial colonization on the implant surface. Clinical trials and extended follow-up periods are essential for validating surface-modified implants' clinical applicability, safety, and long-term outcomes in human patients. This iterative translation process from laboratory experimentation to clinical implementation is crucial for advancing evidence-based practice in implant dentistry and enhancing patient care.

Recent advancements in micro-printing techniques that integrate physical and chemical mechanisms are revolutionizing the creation of micro-patterns on implant surfaces. Researchers can finely tune the microenvironment to enhance cell growth and behavior by combining geometric patterns with customizable sizes and chemical properties.

## Conclusions

Surface modification technologies have emerged as a cornerstone in advancing the osseointegration of titanium dental implants. By incorporating bioactive coatings, these modifications significantly enhance the integration of implants with surrounding bone tissue, ensuring superior biocompatibility and minimizing the risk of foreign body reactions. These coatings mimic natural bone minerals, facilitating direct bonding with living bone and promoting faster healing and improved stability. The ability of bioactive coatings to support cellular adhesion and differentiation further accelerates osseointegration, reducing implant failure rates. Additionally, the customization of coatings, such as the inclusion of peptides like RGD or growth factors like BMP-2, enables targeted therapeutic outcomes, such as enhanced bone formation and sustained implant performance. These advancements translate to long-lasting implants with better load distribution, patient comfort, and aesthetic outcomes. By improving the functionality and durability of implants, surface modification technology elevates oral health and enhances patients' overall quality of life. As this interdisciplinary field continues to evolve, it holds immense potential to transform dental restoration practices worldwide.
